# Tenascin-C regulates CXCR4^+^ B cell migration and cortex formation in the developing bursa of Fabricius

**DOI:** 10.3389/fimmu.2025.1636140

**Published:** 2025-09-04

**Authors:** Ádám Soós, Emőke Szőcs, Viktória Halasy, Csenge Jurenka, Nándor Nagy

**Affiliations:** Department of Anatomy, Histology and Embryology, Faculty of Medicine, Semmelweis University, Budapest, Hungary

**Keywords:** bursa of Fabricius, cortex, B cell development, tenascin-c, CXCL12, CXCR4, chicken

## Abstract

The bursa of Fabricius (BF) is a unique primary lymphoid organ critical for B cell development in its specialized follicular microenvironment. Although the role of the follicular medulla required for B cell maturation is well characterized, the cellular components and function of the ontogenetically later emerging cortex remain less understood. Here, we combined immunocytochemistry, RNAscope, cell culture, and embryo manipulation techniques to investigate the origin and structure of the cortical compartment. Immunostaining of adult BF revealed a heterogeneous B cell distribution in the cortex, with chB6+/CXCR4^high^ cells in the outer region and CXCR4^low/dim^ cells adjacent to the cortico-medullary border. The cortex is supported by CXCL12+/desmin+/vimentin+ mesenchymal reticular cells producing extracellular matrix (ECM), including tenascin-C, which is enriched in the CXCR4^low/dim^ region. Embryonic expression of tenascin-C coincides with the accumulation of CXCR4+ B cell precursors in the presumptive cortical compartment. Functional studies demonstrate that tenascin-C inhibits embryonic CXCR4+ B cell migration, with overexpression disrupting follicle formation. These findings highlight tenascin-C as a key regulator of B cell migration in the embryonic BF and emphasize the importance of a tenascin-C-free mesenchymal environment for the homing of CXCR4^+^ B cell precursors during development. In adults, the complementary expression patterns of tenascin-C and CXCR4 molecules suggest that downregulation of CXCR4 is required for B cell migration through the CXCL12-tenascin-C-rich cortex before exiting the BF.

## Introduction

The bursa of Fabricius (BF) is a primary lymphoid organ in birds responsible for B-lymphocyte maturation (1). The BF is situated dorsal to the colorectum as a diverticulum of the cloaca, which is anatomically connected with the ectodermal-derived portion of the cloacal epithelium (2). In adult chickens, the BF has the size and shape of a chestnut, reaching its maximum size at 10-12 weeks of age (3, 4). The luminal surface exhibits 10-15 longitudinal folds filled with follicles closely associated with the surface epithelium (5). Several histological studies have established that bursal follicles, which are considered the basic structural and functional units of the organ, are composed of a central medullary part divided by a basement membrane from the peripheral cortex (6–8). Morphogenesis of the follicles starts around day 10 during chicken embryonic development when blood-borne myeloid and B cell precursors migrate to the bursa primordium, colonize the epithelial follicle buds, divide intensively, and generate the medullary lymphoid compartment (9–11). The medulla, containing epithelial reticular cells, B cells, macrophages, and dendritic cells provides the bursa-specific microenvironment to support B cell survival, proliferation, maturation, and immunoglobulin (Ig) diversification (2, 10, 12–14). Around hatching, chB6+ B cells migrate through the basement membrane to form the cortical compartment of bursal follicles (7, 15, 16). The cortex of adult lymphoid follicles has morphological and functional properties fundamentally different from those of the medulla; it contains densely packed B cells and macrophages, and, unlike the medulla, the reticular cells develop exclusively from the mesoderm.

Medullary and cortical bursal B cells uniformly express the cell surface chB6 molecule and the BAFF-receptor, also known as the CD268 antigen (17–19). Previous studies have shown that in contrast to the medulla, the cortex contains a rapidly dividing B cell population selectively expressing LT2, CD80, and CXCR4 antigens (15, 20, 21). Furthermore, we and others recently reported that a subpopulation of cortical B cells can be distinguished as clustered around the capillaries based on their expression of chL12 (also known as Ov antigen) and 7H3 surface antigens (22–24), chicken interleukin-2 receptor alpha chain (chCD25) (20), and chicken tumor necrosis factor-like ligand 1 (chTL1A) (25).

Myeloid cells also show a different distribution pattern between the medulla and the cortex. In contrast to TIM4+/Lamp1+ macrophages, the CSF1R+/74.3+/vimentin+ bursal secretory dendritic cells are present only in the medulla, and no occurrence of dendritic cells was reported in the cortex (10, 26, 27). Epithelial reticular cells, forming a complex 3D scaffold for the B cells, are present in the medulla but not the cortex. In contrast, vimentin+/desmin+ mesenchymal reticular cells are characteristic supportive elements of the cortex, which produce extracellular matrix (ECM) proteins, such as collagen I, fibronectin, and laminin (28–31). In primary and secondary lymphoid organs, these ECM molecules outline separate tissue compartments and are crucial to create a 3D scaffold that favors the communication, adhesion, migration, and proliferation of lympho-myeloid cells. It is important to note that the ECM is completely absent in the medulla of bursal follicles (4). In addition to the ECM, stromal cells also produce chemokines and growth factors, through which the cortical stroma may control many cellular processes, including patterning, differentiation, and migration of B cells. *In situ* hybridization and *in vitro* cell migration studies in chicken embryos demonstrated that the CXCL12 chemokine produced by mesenchymal-derived reticular cells and its CXCR4 receptor are essential for both populating the bursa primordium with B cell precursors and emigration of medullary B cells to the follicular cortex (21, 32). Elegant experiments have shown that BF-derived B cells emigrating to the peripheral lymphoid organs originate directly from the cortex of lymphoid follicles (33–35), but the mechanism that controls cortical B cell migration is not fully characterized. It is suggested that B cells leave the BF through the cortical capillaries, and this process is strongly related to the expression level of the CXCR4 receptor.

In this study, we performed electron and light microscopy to characterize the cellular and extracellular composition of the cortical region of bursal lymphoid follicles. We found that, among the complex ECM proteins uniformly expressed in the cortex and interfollicular connective tissue, tenascin-C is highly specific to the inner region of the cortex. Using *in vitro* organ culture and cell migration assays, we demonstrate that tenascin-C acts as an inhibitory ECM molecule for B cell migration. This inhibitory role was further validated *in vivo* using the retrovirus-mediated overexpression of Sonic hedgehog (Shh), which induced ectopic tenascin-C expression in the BF mesenchyme, inhibiting bursal follicle formation. These findings highlight the critical role of tenascin-C exclusion in the embryonic bursal mesenchyme for the proper homing of CXCR4-expressing B cell precursors. In addition, polarized expression patterns of CXCL12-CXCR4 and tenascin-C in the adult BF support the idea that B cells must downregulate their CXCR4 expression to exit the CXCL12-tenascin-C rich stromal microenvironment of the BF follicular cortex to colonize peripheral organs.

## Materials and methods

### Animals

Fertilized White Leghorn (*Gallus gallus domesticus*), SPF chicken eggs were obtained from commercial breeders (Prophyl-BIOVO Ltd., Hungary). The eggs were incubated at 37.5°C in a humidified incubator (Heka 1+ incubator Brutgerate, TS-7000C Rietberg, Germany) at 90% humidity, and the age of the embryos was determined by the number of embryonic (E) days. Hatched animals were housed in the aviary of the Department of Anatomy, Histology, and Embryology, Semmelweis University. All animal work was conducted according to relevant national and international guidelines and approved by the Animal Care and Use Committee at Semmelweis University, Budapest, Hungary.

### Immunocytochemistry

For cryosections, tissue samples were fixed in 4% paraformaldehyde (PFA) for 1 hour at room temperature, then infiltrated with 15% sucrose overnight at 4°C, followed by 7.5% gelatine (Sigma, G-2625) in 15% sucrose for 1 hour at 37°C. Gelatine-impregnated tissues were rapidly frozen at −50°C in 2-methylbutane (Sigma, 78-78-4). 12 μm thick cryosections were labelled with primary antibodies (**Table 1**) for 1 hour, followed by biotinylated secondary antibodies (**Table 2**) (Vector Laboratories) and avidin-biotinylated peroxidase complex (Vectastain Elite ABC kit, Vector Laboratories, PK-6100). Endogenous peroxidase activity was quenched with 3% hydrogen peroxide (Sigma, H1009) for 10 minutes. The binding sites of the primary antibodies were visualized by 4-chloro-1-naphthol (Sigma, C8890).

**Table 1:** List of primary antibodies.

Antigen	Clone	Source	Isotype
collagen I	SPID8	Developmental Studies Hybridoma Bank (DSHB)	mouse IgG_1_
collagen III	3B2	DSHB	mouse IgG_1_
collagen IV	IIB12	DSHB	mouse IgG_1_
collagen VI	5C6	DSHB	mouse IgG_1_
collagen XVIII	6C4	DSHB	mouse IgG_1_
Laminin	3H11	DSHB	mouse IgG_1_
Agrin	6D2	DSHB	mouse IgG_1_
Fibronectin	B3/D6	DSHB	mouse IgG_1_
Fibrillin	JC3	DSHB	mouse IgG_1_
Perlecan	5C9	DSHB	mouse IgG_1_
Versican	12C5-s	DSHB	mouse IgG_1_
Podocalyxin	MEP21	Dr. Kelly McNagny, Canada (36)	mouse IgG_1_
Vimentin	3B4	DSHB	mouse IgG_2A_
Desmin	DE-R-11	Abcam (ab28026)	mouse IgG_1_
tenascin-C	M1-B4	DSHB	mouse IgG_1_
Cytokeratin	Lu-5	Millipore (MAB3406)	mouse IgG_1_
Shh	5E1	DSHB	mouse IgG_1_
avian myeloblastosis virus, p19^(gag)	3C2	DSHB	mouse IgG_1_
CXCR4	9D9	Dr. Sonja Hartle, BioRad (MCA6012GA)	mouse IgG_2A_
chB6 (Bu-1a/b)	BoA1	BioRad (19)	mouse IgG_1_

**Table 2:** List of secondary antibodies.

Secondary antibodies	Source	Catalog number
biotinylated anti-mouse IgG (H+L)	BioMarker Kft.	BA-9200
biotinylated anti-mouse IgM	BioMarker Kft.	BA-2020
biotinylated anti-rabbit IgG	BioMarker Kft.	BA-1000
Alexa Fluor 488 donkey anti-mouse IgG	Thermo Fisher Scientific	A-21202
Alexa Fluor 594 donkey anti-mouse IgG	Thermo Fisher Scientific	A-21203
Alexa Fluor 594 goat anti-mouse IgG_2A_	Thermo Fisher Scientific	A-21135
Alexa Fluor 647 donkey anti-mouse IgG	Thermo Fisher Scientific	A-31571

### Immunofluorescence staining

12 μm-thick cryosections were stained with primary antibodies listed in **Table 1** for 1 hour, followed by fluorescently labelled secondary antibodies (**Table 2**) for 45 minutes. To assess cell proliferation, 10 µM 5-ethynyl-20-deoxyuridine (EdU) was added to the culture medium 3 hours before fixation with 4% paraformaldehyde (PFA). EdU incorporation was subsequently detected using the Click-iT EdU Imaging Kit (Thermo Fisher, C10337). Cell nuclei were stained with 4,6-diamidino-2-phenylindole (DAPI, Vector Laboratories), and sections were covered by aqueous Poly/Mount (Polyscience Inc., 18606). Sections were examined with fluorescent (Nikon Eclipse E800), confocal scanning (Zeiss LSM 780) and STED (Abberior Expert Line- Nikon Ti2) microscopy; section images were recorded using CellSens and ZEISS ZEN Imaging proprietary softwares. Image processing was performed using ImageJ and Adobe Photoshop CC 2020 programs.

### RNAscope *in situ* hybridization

4-week-old chicken bursa of Fabricius was fixed in 4% PFA for 24 hours at 4°C, washed in PBS and infiltrated with 10% sucrose overnight at 4°C, followed by 20% and 30% sucrose solutions. Tissues were impregnated with 7.5% gelatine for two hours at 37.5°C. Gelatine-impregnated tissues were rapidly frozen at −50°C in 2-methylbutane. 12 µm thick cryosections were collected on Epredia™ Superfrost Plus Adhesion Slides (Fisher Scientific, J1800AMNZ) and stored at -80°C until use. RNAscope was performed using the RNAscope Fluorescent Multiplex Detection Reagent Kit v2 (Bio-Techne, 323136) according to the manufacturer’s protocol. Hybridization was performed using the chicken-specific Gg-CXCL12-C2 probe (Bio-Techne, 458251). In case of double labelling, the RNAscope method was done first, followed by immunocytochemistry.

### Electron microscopy

4-week-old chicken bursa of Fabricius were fixed in 4% buffered glutaraldehyde for 24 hours at 4°C, washed 3 times for 5 minutes in Milloning buffer (830ml 2.26% Na2HPO4 • 2H2O- Merck, 106462 and 170ml 2.52% NaOH - Merck, 1.06346.1000), then post-fixed with 1% osmium tetraoxide for 2 hours (Merck - B000970). Tissue samples were dehydrated in graded ethanol, placed in propylene oxide (Merck, 12492) twice for 5 minutes, followed by propylene oxide and EPON (Araldite Resin Grade 6005 - EMS-10920; Poly-bed 812 - EMS-14900; DDSA-Dodenyl Succinic Anhydride- EMS-13710; NMA-Nadic Methyl Anhydride-EMS-19000; DMS 30-2,4,6-Tris Dimethylaminomethylphenol-EMS-13600). Tissue samples were embedded in Polybed/Araldite 6500 (Polysciences Inc.) oriented and polymerized at 56°C for 2 days. 50-70 nm ultra-thin sections were contrasted with a mixture of uranyl acetate (10 min) and lead citrate (12 min). Sections were examined with a Jeol JEM-1200EX transmission electron microscope.

### *In vitro* B cell migration assay

For the embryonic B cell migration assay, bursa of Fabricius was isolated from E13 chick embryos and cultured in the presence of CXCL12 (100 ng/ml; R&D Systems, 6448-SD-025) in RPMI 1640 (Sigma, R8758) culture media. To determine how different matrix molecules affect B cell migration, we coated Petri-dishes with 10 μg/ml laminin (Sigma, L2020), 10 μg/ml tenascin-C (Sigma, CC115) or 20 μg/ml fibronectin (Sigma, F1141) diluted in RPMI, containing 5% Penicillin/Streptomycin (Pen/Strep, Sigma, P0781) at 37°C for 2 hours. Petri-dishes were washed twice with PBS, and E13 bursal folds (n=6/experimental setup) were explanted on the different ECM-coated surfaces. Organ cultures were maintained at 37°C, 5% CO_2_ for 24 hours.

### B cell adhesion assay

For the B cell adhesion assay, BF was dissected from E18 chicken embryos and enzymatically digested in RPMI 1640 medium (Sigma, R8758-500ML) containing 0.03% collagenase IX (Sigma, C9722) and 2% dispase II (Stem Cell Technologies, 07913) for 30 minutes at 37°C with gentle agitation. The resulting cell suspension was filtered through a 40 µm cell strainer, washed twice in RPMI supplemented with 10% FBS (fetal bovine serum, Capricorn Scientific, FBS-12B) and 5% Penicillin/Streptomycin. 35 mm Petri dishes were coated for 1 hour at 37°C with chicken tenascin‐C (10 µg/ml) and CXCL12 (100 µg/ml; PeproTech, 250-20A). Double-coated dishes were washed twice with PBS, and BF cells were seeded at a density of 2 × 10^5^ cells per dish and incubated at 37°C, 5% CO_2_. Inhibition of the CXCR4 receptor was achieved by adding the CXCR4-antagonist AMD3100 at a concentration of 200 µM (Sigma, 239820). Control cells were cultured in RPMI. After incubation for 6 hours, non-adhesive cells were removed by gentle aspiration using a pipette, the adhered cells fixed in ice-cold 100% methanol for 20 minutes and anti-chB6 immunostaining was performed. All cell adhesion assays were repeated 3 times.

### *Shh*-RCAS retrovirus-mediated perturbation and chorioallantoic membrane transplantation

We used replication-competent avian retrovirus (RCAS) expressing chick Shh (37) to induce ectopic expression of tenascin-C (38). Shh misexpression in the developing chick bursa of Fabricius was performed following the method of Nagy et al. (21, 39). Briefly, 1 μl Shh-RCAS virus was injected into the mesenchymal layer of the bursa of Fabricius of E9 chicken embryos using a Hamilton syringe attached to a Narishige brand microinjector. Shh-RCAS injected E9 bursa primordia were transplanted onto the chorioallantoic membrane of E9 chicken embryos and cultured for 8 days. CAM grafts were excised and fixed in 4% paraformaldehyde for 1 hour at RT, embedded in gelatin for cryosectioning and analyzed for Shh and tenascin-C expression. 3C2 immunocytochemistry was used to detect the presence of the viral vector. Control grafts were injected with PBS. At least three replicates were collected and analyzed for each experimental condition.

### Statistical analysis

Statistical analysis was performed using the Kruskal-Wallis test or ANOVA with a *post-hoc* Dunn’s test or Tukey test (R Core Team). P-values were adjusted with Holm correction. P<0.05 was considered significant, and the following significance levels were used: **p<0.01; ***p<0.001; ****p<0.0001. Error bars represent the standard error of the mean.

## Results

### Expression of CXCL12 and desmin defines the cortex of adult lymphoid follicles in the chicken bursa of Fabricius

**Figure 1:**
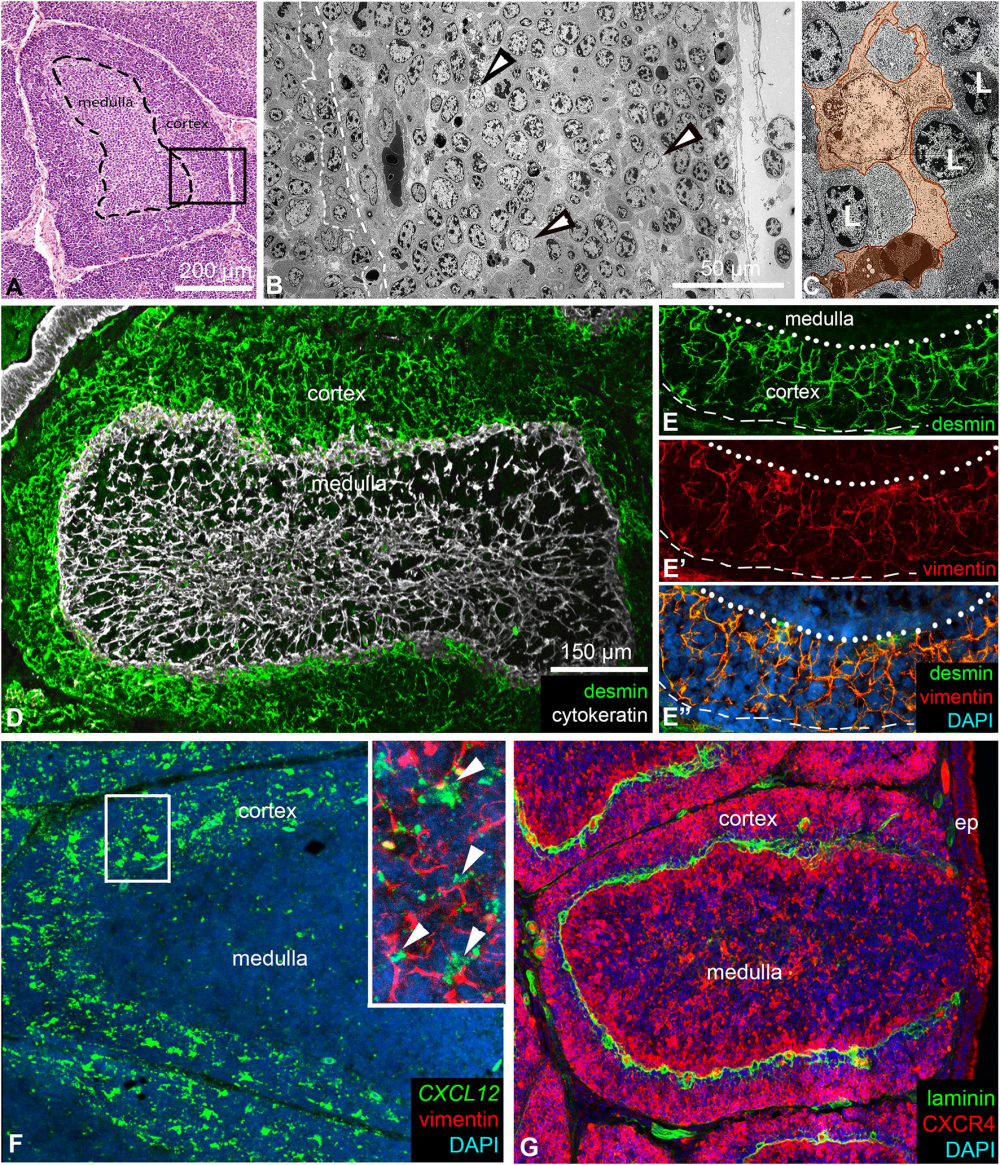
Structure of lymphoid follicles in the chicken bursa of Fabricius. **(A)** Haematoxylin-eosin stained paraffin-embedded section of an 8-week-old chicken bursa of Fabricius. Each bursal follicle contains a central medullary and an outer cortical region, separated by a basement membrane (dashed line). **(B)** Electron micrograph of the cortex (see the outlined area in **A)** shows several reticular cells (arrowheads) scattered among cortical B cells. The outlined area marks the cortico-medullary epithelium. **(C)** The euchromatic nuclear structure of reticular cells (orange) differs from the heterochromatic nuclear structure of lymphocytes. **(D)** Reticular cells of the medulla are of epithelial origin and express cytokeratin intermediate filaments. In contrast, reticular cells of the cortex are of mesenchymal origin and are desmin+. **(E, E’, E’’)** The dotted line indicates the cortico-medullary border; the dashed line marks the outer border of the cortex. Desmin+ mesenchymal reticular cells co-express vimentin intermediate filaments. **(F)** Desmin+/vimentin+ reticular cells in the cortex uniformly express the CXCL12 chemokine ligand shown by *in situ* hybridization. Magnified view in the inset. Arrowheads show CXCL12+/vimentin+ cortical reticular cells. **(G)** B cells within the cortex are CXCR4+. Laminin marks the basement membrane under the cortico-medullary border and around cortical capillaries.

Avian B cell development occurs within the specialized microenvironment of the lymphoid follicles of the bursa of Fabricius. While the stromal cells of the follicular medulla have been extensively characterized (4, 7, 40), the specific roles of resident cell populations and the cellular microenvironment of the cortex, which emerges later during ontogeny, remain largely unknown. Histological analysis of 8-week-old chicken bursal sections shows the follicular medulla as a central, loosely packed region with large lymphocytes, while the cortex surrounds the medulla as a dense, outer compartment. Hematoxylin and eosin staining highlights this clear distinction, with the cortex showing darker staining due to its higher cell density (**Figure 1A**). A thin connective tissue layer separates the individual follicles. Electron microscopy further highlights that the cortex contains numerous lymphocytes localized between cortical reticular cells (**Figure 1B**). The cortical reticular cells were identified by their prominent euchromatic nuclei and multiple cellular processes (**Figure 1C**).

In our previous studies addressing the origin of medullary stromal cells, we described that ontogenetically the medulla and the cortex have different origins (2, 41). Medullary epithelial cells, which form the supportive reticular network, are derived from the ectoderm and express cytokeratin intermediate filaments (**Figure 1D**). In contrast, cortical reticular cells arise from the mesoderm and express both desmin and vimentin intermediate filaments (**Figures 1D, E-E**). Importantly, while desmin is a specific marker for cortical reticular cells, vimentin is expressed in both cortical reticular cells and the medullary bursal secretory dendritic cells (BSDCs) (42). RNAscope *in situ* hybridization showed that *CXCL12* expression is restricted to the cortex and is specifically produced by vimentin immunoreactive cortical reticular cells (**Figure 1F**). Recently, we have demonstrated that cortical chB6+ B cells selectively express the CXCR4 receptor (21, 32). Double immunofluorescence shows that CXCR4^high^ B cells in the cortex are restricted to the outer cortical region while CXCR4^low/dim^ B cells are localized near the cortico-medullary border, concentrated around laminin immunoreactive cortical capillaries (**Figure 1G**). The laminin+ basement membrane separates the cortex from the medulla at the cortico-medullary border.

### Cortical reticular cells produce multiple extracellular matrix proteins

**Figure 2:**
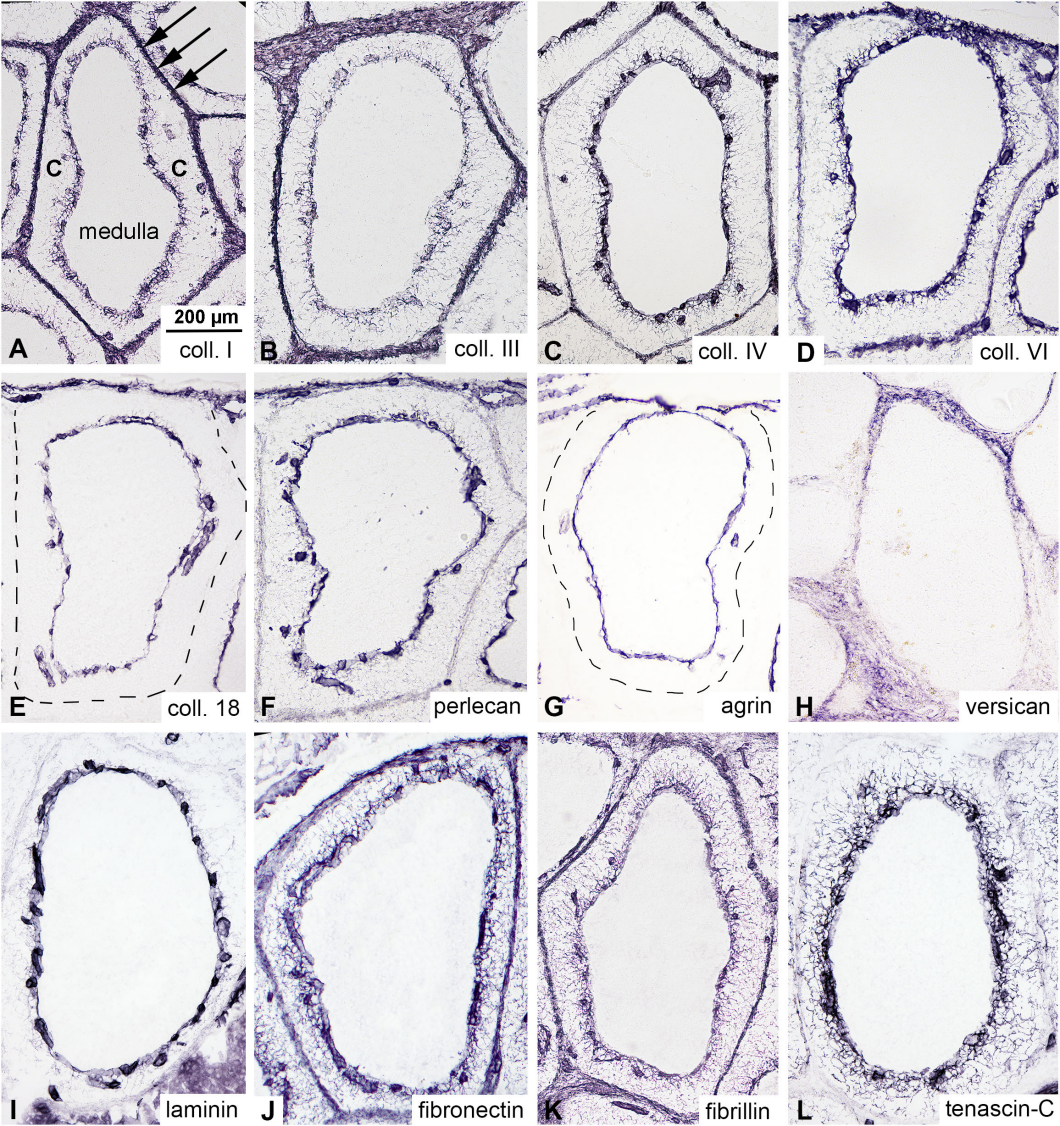
Expression of extracellular matrix (ECM) proteins in the cortex of the bursal follicles. Immunocytochemistry was performed on consecutive sections of the adult chicken bursa of Fabricius to examine the ECM expression pattern in bursal follicles. **(A-D)** Collagens are highly expressed in the cortex of the follicles and interfollicular connective tissue septum (arrows). There are no collagens inside the medulla. **(E-G)** Heparan-sulphate proteoglycans (collagen type 18, perlecan and agrin) are specifically expressed in the basement membrane of the cortico-medullary epithelium and cortical endothelial cells, respectively. **(H)** Versican, a chondroitin-sulphate type proteoglycan, shows concentrated interfollicular connective tissue immunoreactivity. Immunostaining of glycoproteins indicates that **(I)** laminin is specifically expressed in the basement membrane. **(J)** Fibronectin and **(K)** fibrillin are diffusely distributed throughout the cortex and interfollicular connective tissue, while **(L)** tenascin-C shows intense fibrillar staining concentrated in the inner region of the cortex.

Although collagen III, fibronectin, and laminin are known to be expressed in the cortex of bursal follicles (4, 29) a detailed analysis of extracellular matrix (ECM) protein expression has not been performed. In order to characterize the immunophenotype of the ECM produced by cortical reticular cells, comparative immunostainings were performed using a panel of commercially available collagen-, proteoglycan-, and glycoprotein-specific markers. All major ECM molecule families are highly expressed within the bursa, with distinct expression patterns. Collagens (type I, III, IV, and VI) are expressed by the interfollicular connective tissue septae, the basement membrane of the cortico-medullary epithelium, and the basement membrane of endothelial cells of cortical capillaries (**Figures 2A–D**). Heparan-sulphate proteoglycans (collagen XVIII, perlecan, and agrin) are specifically expressed in basement membranes (**Figures 2E–G**), while versican, a chondroitin-sulphate proteoglycan is restricted to the interfollicular connective tissue (**Figure 2H**). Among glycoproteins, laminin expression is restricted to basement membranes (**Figure 2I**), while fibronectin and fibrillin are highly expressed in the cortex, showing intense fibrillar staining from the cortico-medullary border to the interfollicular connective tissue (**Figures 2J, K**). In contrast, tenascin-C expression is restricted to the cortex, showing strong immunoreactivity in the inner cortical region and a gradient-like expression pattern from the cortico-medullary border towards the interfollicular septae. Compared to other ECM proteins, tenascin-C was not expressed in the interfollicular connective tissue (**Figure 2L**).

### Complementary expression patterns of tenascin-C and CXCR4 molecules in the follicular cortex

**Figure 3:**
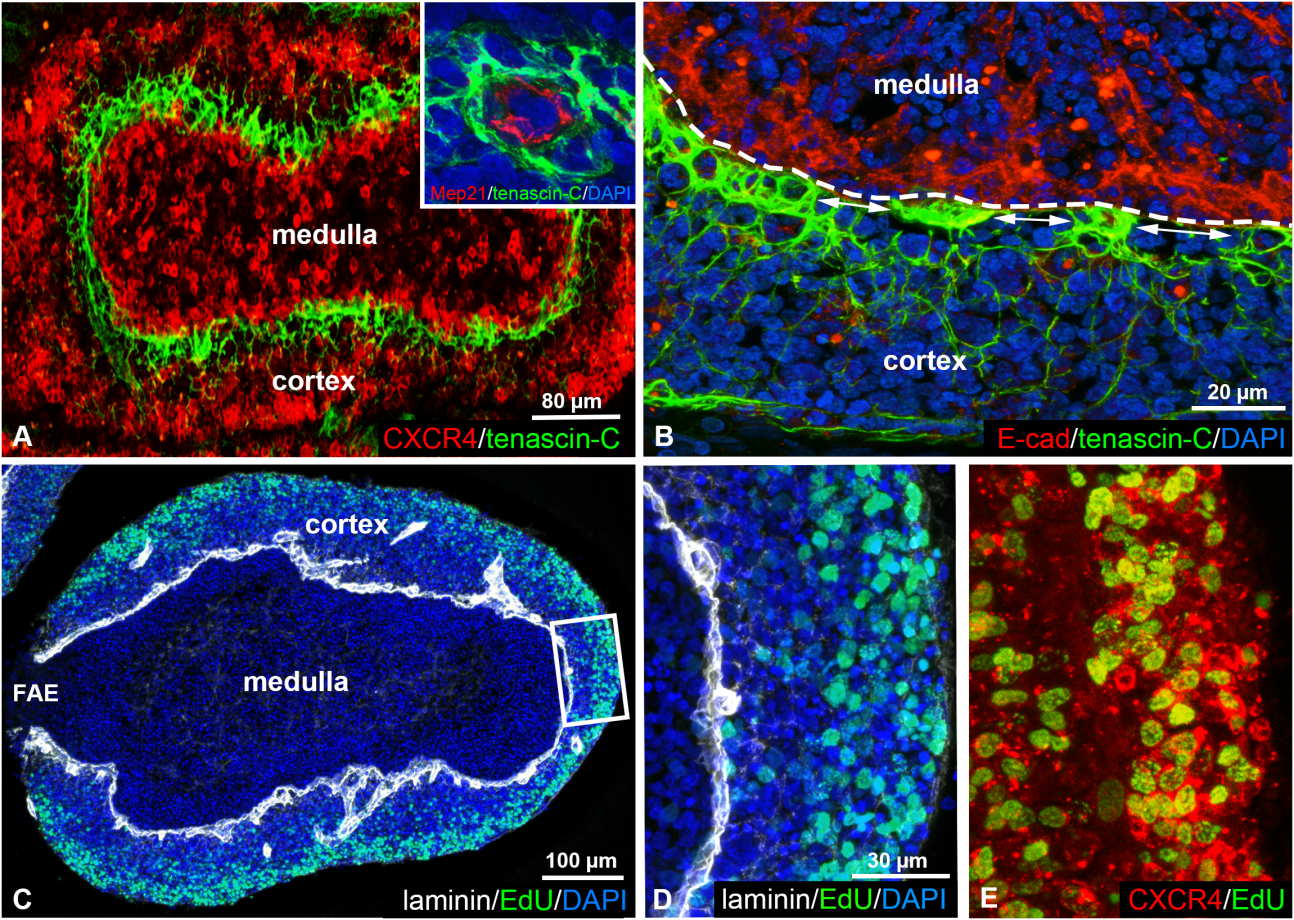
Tenascin-C and CXCR4 show a complementary expression pattern in the follicular cortex. **(A, B)** Double immunostaining of tenascin-C and CXCR4 in the adult bursal follicle shows robust tenascin-C expression in CXCR4^low/dim^ regions along the cortico-medullary border (dashed line). Tenascin-C immunoreactivity is highest around cortical capillaries (inset) at the E-cadherin+ cortico-medullary epithelium **(B)** and gradually decreases between the capillaries (double arrows) and towards outer cortical regions. **(C-E)** EdU incorporation reveals that CXCR4^high^ B cells within the outer region of the cortex are highly proliferative. Laminin expression marks the basement membrane along the cortico-medullary border and around cortical capillaries.

Recently, we demonstrated that subpopulations of chB6+ cortical B cells can be distinguished by their selective expression of the CXCR4 receptor (21). Immunostaining of adult chicken BF shows that CXCR4^high^ B cells are concentrated in the outer cortical region while CXCR4^low/dim^ B cells are localized near the cortico-medullary border (**Figure 3A**). The expression of tenascin-C in the cortex exhibits a complementary pattern, with its gradient highest around cortical capillaries (**Figure 3A** inset) near the E-cadherin immunoreactive cortico-medullary epithelium and gradually decreases towards the outer cortical regions (**Figures 3A, B**). Interestingly, alternating with the tenascin-C-rich capillary region, gaps with low expression level of tenascin-C can also be observed in the inner cortical environment (**Figure 3B**).

Previous studies demonstrated that B cells leave the bursa directly from the follicular cortex through cortical capillaries and that bursal emigrants originate from the rapidly dividing cortical B cells (33–35). EdU incorporation confirmed the presence of proliferating cells within the cortex, CXCR4^high^ cortical cells representing highly proliferative regions, compared to low proliferative CXCR4^low/dim^ cells in tenascin-C rich zones adjacent to the cortico-medullary border (**Figures 3C–E**).

### Tenascin-C is not expressed by the embryonic bursa mesenchyme

In recent years, several aspects of embryonic bursal cell migration have been uncovered, highlighting the role of the mesenchymal-derived CXCL12 chemokine in guiding CXCR4+ B cell precursors to colonize the developing lymphoid follicles (21, 32, 43). In addition to CXCL12 signaling, it has been reported that the ECM plays a crucial role in coordinating cell migration through ECM-cell and ECM-chemokine interactions. Although fibronectin is known to be expressed in the embryonic bursa mesenchyme (29), a detailed analysis of ECM expression patterns during bursa of Fabricius development has not been performed.

**Figure 4:**
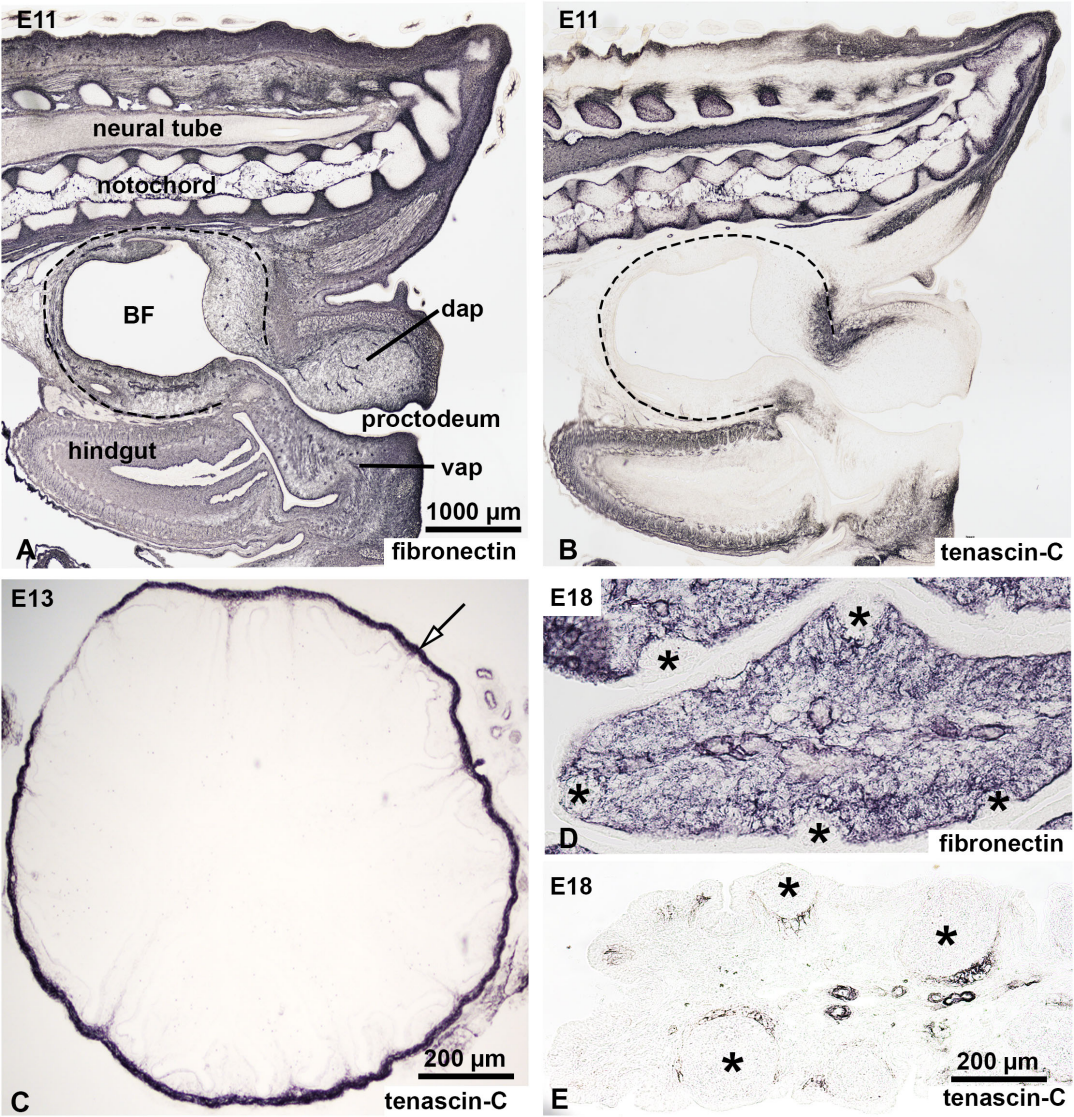
Expression of fibronectin and tenascin-C in the developing chick bursa of Fabricius. Expression of fibronectin and tenascin-C was analyzed in the developing BF at E11 **(A, B)**, E13 **(C)** and E18 **(D, E)**. **(A, B)** Sagittal sections through the tail bud of an E11 embryo show the expression patterns of fibronectin and tenascin-C. Dashed lines mark the BF. **(A)** Anti-fibronectin immunostaining reveals robust expression in the extracellular matrix of the tail bud, the BF, and the hindgut mesenchyme. **(B)** Tenascin-C expression, in contrast, is restricted to the vertebral column, tip of the tail bud and hindgut mesenchyme, with no detectable expression in the BF at this stage. **(C)** Transverse section of the BF at E13 stained with anti-tenascin-C. At this stage, tenascin-C is absent in both the lymphoid compartment and the surrounding connective tissue of the BF. However, the connective tissue capsule of the BF (arrow) shows strong tenascin-C immunoreactivity. **(D, E)** Longitudinal section of the BF fold at E18. **(D)** Fibronectin staining demonstrates uniform expression in the mesenchymal compartment but is notably absent in the developing lymphoid follicles (asterisks). **(E)** Tenascin-C immunoreactivity emerges at E18, specifically in the mesenchymal cells surrounding the developing follicles. BF, bursa of Fabricius; dap, dorsal anal lip; vap, ventral anal lip.

In chicken embryos, colonization of the bursal epithelial-mesenchymal rudiment by chB6+/CXCR4+ B cell precursors occur within a restricted developmental window between embryonic days 10 and 15 (E10-E15). To investigate the role of ECM proteins during this critical stage, we examined their distribution at E11, when the first wave of chB6+ B cells enters the bursal mesenchyme. At this developmental stage, the BF mesenchyme shows intense fibronectin immunoreactivity (**Figure 4A**). ECM-specific immunostaining on consecutive sections confirmed that all tested collagens, proteoglycans, and glycoproteins are broadly expressed in the bursal primordium with the exception of tenascin-C (**Figure 4B**). Basement membranes are specifically labeled by laminin, agrin, and collagen IV (data not shown).

The earliest sign of tenascin-C expression is seen in the connective tissue capsule of the BF primordium at developmental stage E13 (**Figure 4C**). At E18, as bursal development progresses and B cell colonization is completed, the outer mesenchymal compartment of developing bursal follicles shows intense fibronectin expression, but the epithelial follicle buds are fibronectin negative (**Figure 4D**). In contrast, tenascin-C expression is localized in the mesenchymal region surrounding larger developing follicles corresponding to the prospective cortical region, which begins to develop shortly after hatching (**Figure 4E**). Expression of tenascin-C around E17-E18 correlates with the loss of the follicle bud colonization potential of bursal B cell precursors (2).

### Overexpression of tenascin-C inhibits B cell colonization during chicken BF development

**Figure 5:**
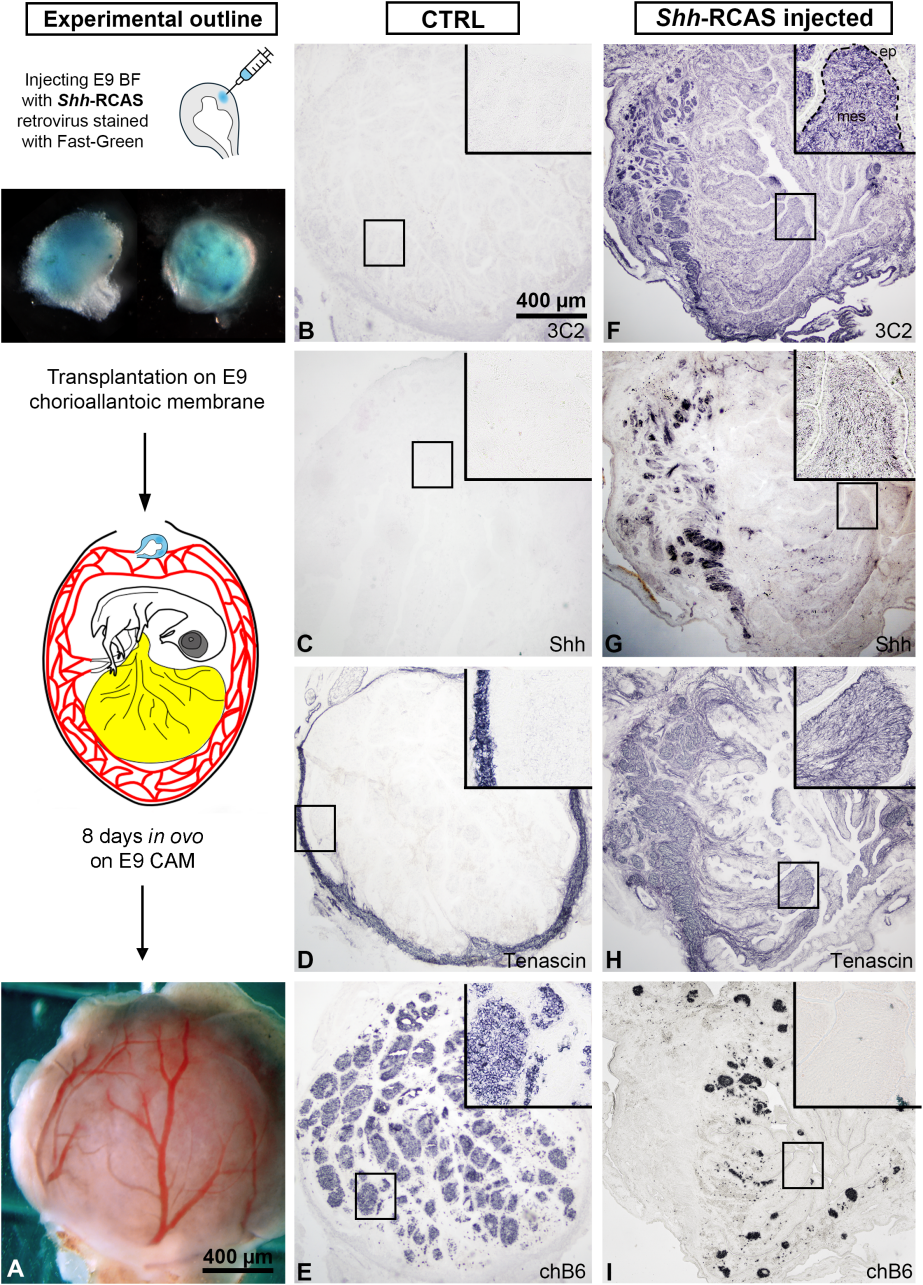
RCAS virus-mediated overexpression of Shh induces ectopic tenascin-C expression in the embryonic bursa mesenchyme, leading to abnormal lymphoid follicle formation. **(A)** Schematic illustration depicts the experimental setup, where dissected E9 bursa of Fabricius was injected *ex vivo* with RCAS-Shh and cultured *in ovo* for 8 days on an E9 chick chorioallantoic membrane (CAM). Serial cross-sections from control CAM grafts were immunostained for the RCAS virus-specific 3C2 antibody **(B)**, Shh **(C)**, tenascin-C **(D)**, and chB6 **(E)**. Magnified views of outlined bursal folds are presented in insets. **(F)** In *Shh*-RCAS injected grafts, robust RCAS expression was detected throughout the bursa mesenchyme using the 3C2 antibody, indicating effective viral transduction and ectopic Shh protein expression. **(G)** Shh expression was localized to the bursa mesenchyme, confirming successful overexpression. **(H)** Overexpression of Shh significantly upregulated tenascin-C expression within the bursa mesenchyme. **(I)** The ectopic expression of tenascin-C was associated with abnormal lymphoid follicle formation, characterized by a reduction in chB6+ B cell precursors.

To determine whether tenascin-C influences B cell migration *in vivo*, we performed embryo manipulation to experimentally modify the expression of tenascin-C within the bursal mesenchyme. Recent studies have uncovered signaling pathways that coordinate the expression pattern of mesenchymal growth factors and extracellular matrix proteins during vertebrate development. Sonic hedgehog (Shh) is an important morphogen that orchestrates key processes in the vertebrate nervous system, somite, gastrointestinal tract and limb development (39, 44–46). Importantly, Shh is not expressed in the BF at any developmental stage (2). According to Ting-Berreth et al., experimental activation of the Shh signaling in the mesenchyme of chicken feather buds, results in ectopic tenascin-C expression (38). Building on this data, we used the replication-competent retroviral vector (RCAS) system to experimentally overexpress Shh within the bursa mesenchyme to assess how ectopic expression of tenascin-C affects embryonic B cell migration. E9 bursa primordia were microinjected with RCAS-Shh retrovirus, transplanted onto the chorioallantoic membrane (CAM) of E9 chicken embryos and cultured for an additional 8 days *in ovo* (**Figure 5A**). Control bursa grafts showed normal B cell colonization, characterized by well-developed chB6+ lymphoid follicles along the bursal folds. In these CAM grafts, Shh expression was absent, and tenascin-C expression was restricted to the bursal connective tissue capsule (**Figures 5B–E**). In contrast, RCAS-Shh injected grafts showed effective viral infection of the bursal mesenchyme, as confirmed by anti-3C2 immunostaining specific for the *gag* protein of RCAS virus (**Figure 5F**), supporting the robust Shh protein expression in the bursal mesenchyme (**Figure 5G**). The ectopic activation of Shh signaling resulted in the mesenchymal expression of tenascin-C (**Figure 5H**), associated with disrupted B cell colonization and only rudimentary chB6+ lymphoid follicle development (n=6, **Figure 5I**). These findings suggest that the exclusion of tenascin-C from the embryonic bursal mesenchyme is essential for the proper colonization of B cells and the development of lymphoid follicles in the BF.

### Tenascin-C inhibits *in vitro* migration of embryonic B cells

**Figure 6:**
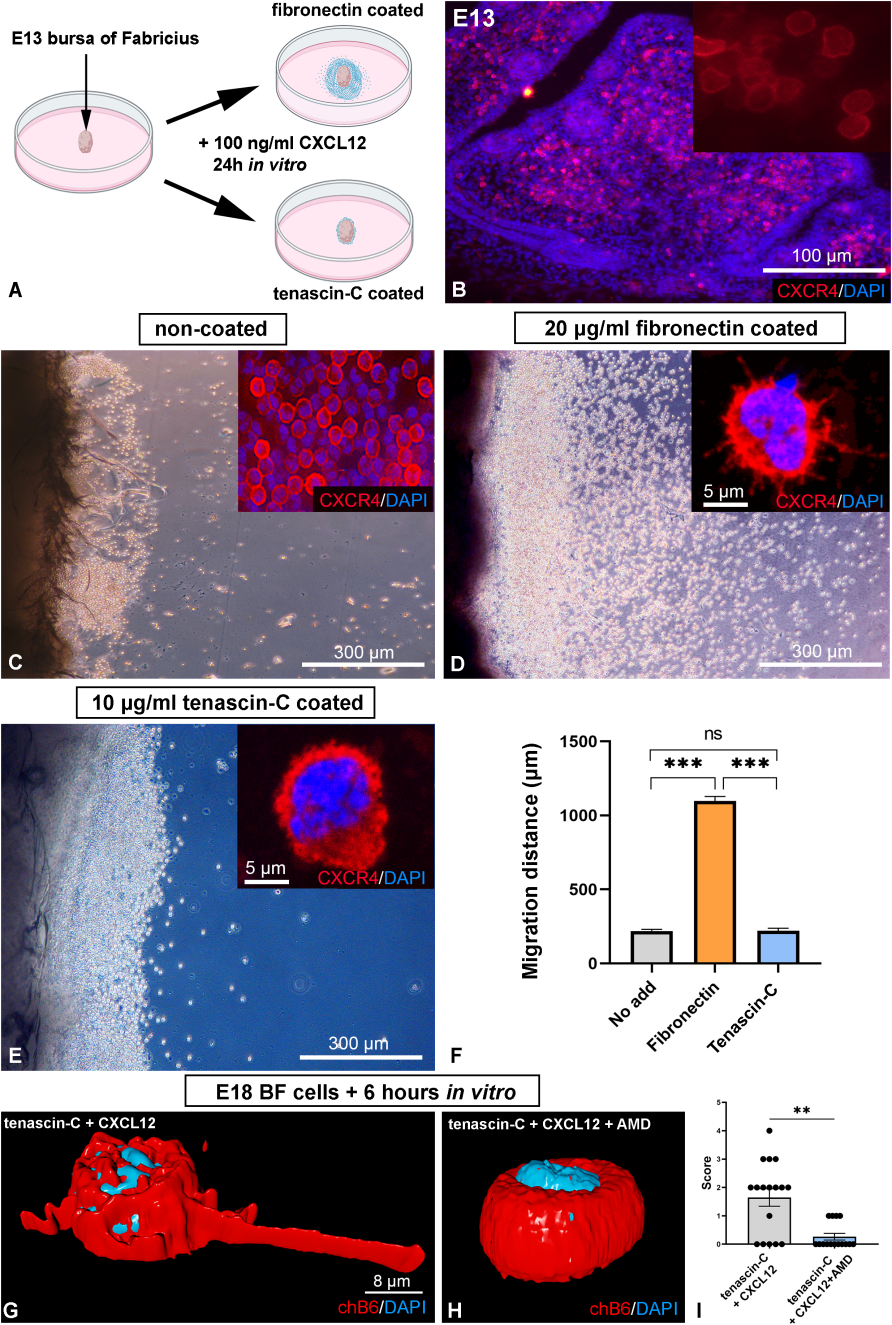
Effect of tenascin-C and tenascin-C bound CXCL12 on B cell migration and adhesion. **(A)** Schematic illustration of embryonic BF organ culture experiments. **(B)** E13 chicken bursal folds containing CXCR4+ B cells were used to study cell migration induced by the CXCL12 chemokine. **(C)** CXCR4+ bursal cells show limited migration on untreated plastic cell culture dish surface in response to 100 ng/ml CXCL12. The inset shows a magnified view of CXCR4+ cells (red). DAPI (blue) was used for nucleus staining. **(D)** Coating the plastic surface with 20 µg/ml fibronectin induces robust CXCR4+ cell migration from the explant. Note the presence of extensive microvilli and filopodia on the surface of a CXCR4+ cell (inset). **(E)** In contrast, the presence of tenascin-C markedly inhibits CXCL12-induced cell migration, the CXCR4+ cells were spherical (inset). **(F)** Quantitative analysis of the migration distance of CXCR4+ cells for each experimental group. Data presented with the standard error of the mean. **(G)** CXCR4+/chB6+ B cells isolated from E18 BF and cultured on the surface of CXCL12 immobilized to tenascin-C induced polarization of B cells with long filopodia, **(H)** which is effectively blocked by treatment with the CXCR4 receptor antagonist AMD3100, where B cells were spherical. **(I)** Quantitative analysis of cell morphology of bursal B cells for each experimental group. The length of filopodia of chB6+ cells of 20 different high-power (200×) fields was counted in untreated (RMPI-1640 culture medium) and AMD3100-treated cultures. chB6+ cells with filopodia longer than the cell diameter were scored. These are the representative images of the morphologic phenotypes detected in 3 separate experiments, including screening of at least 200 cells in each experiment. Significance levels: **p<0.01, ***p<0.001, ns, not significant. Error bars represent the standard error of the mean.

To confirm the inhibitory effect of tenascin-C on B cell precursor migration, E13 BF folds were cultured on fibronectin or tenascin-C coated plastic surfaces in the presence of CXCL12 (**Figure 6A**), a chemokine known to induce robust migration of bursal B cells (21). At this developmental stage, large number of CXCR4-expressing B cells are localized within the bursal mesenchyme (**Figure 6B**). When 100 ng/ml CXCL12 was added to the culture medium, limited migration of CXCR4+ cells was observed on non-coated Petri dishes (**Figure 6C**). Coating the surface with 20 µg/ml fibronectin significantly enhanced migration, as indicated by the pronounced movement of CXCR4+ cells away from the explants and the appearance of numerous membrane protrusions on their cell surface (**Figure 6D**, inset). In contrast, a 10 µg/ml tenascin-C coating markedly inhibited cell migration, with CXCR4+ cells exhibiting round morphology and a reduced migratory distance (220.6 ± 17.81 µm) compared to fibronectin (1098 ± 29.63 µm) (**Figures 6E, F**). Despite the initial response to CXCL12, characterized by the emigration of CXCR4+ cells from the explants in all conditions, further migration was effectively blocked on tenascin-C-coated surfaces. Morphological analysis revealed that cells on the tenascin-C surface adopted a round, non-polarized morphology (**Figure 6E**, inset). We conclude that the presence of tenascin-C is inhibitory for B cell migration.

### Tenascin-C-bound CXCL12 induces filopodia in B cells

At the time when pre-bursal B cells stop colonizing the developing lymphoid follicles, tenascin-C expression appears around the follicle buds (**Figure 4E**). Around hatching, the pattern of CXCL12 expression changes: both molecules are strongly produced in the developing cortex. In the fully mature bursal follicles, tenascin-C and CXCL12 are exclusively detected within the cortex (**Figures 1F**, **3A, B**). Because tenascin-C can effectively bind CXCL12 through conserved motifs (47, 48), we also analyzed the effect of CXCL12 in immobilized form adsorbed sequentially onto the tenascin-C coated surface. After 6 hours of culture, we observed that E18 bursal cells plated onto tenascin-CXCL12 double-coated dishes displayed two distinct morphologies depending on CXCR4 signaling activity. In the presence of culture media with no additives, B cells adopted an elongated morphology, with long cell protrusions (filopodia) spreading along the CXCL12+tenascin-C double-coated surface (**Figures 6G, I**). However, inhibition of CXCR4 using 200 µM CXCR4 antagonist AMD3100 abolished CXCL12-induced polarization, and B cells exhibited round, non-polarized morphology (**Figures 6H, I**) similar to B cell precursors analyzed in the migration assay (**Figure 6E**). Immunostaining with the chB6 antibody confirms their relative morphology in the tenascin-CXCL12 double-coated surfaces. These findings highlight the importance of ECM components such as tenascin-C in modulating CXCL12 signaling and suggest a potential mechanism by which the extracellular environment influences B cell behavior during BF development.

## Discussion

The bursa of Fabricius (BF) is a highly specialized primary lympho-epithelial organ essential for avian B cell development. Its lymphoid follicles, consisting of a medulla and a cortex, provide specific microenvironments that regulate the proliferation, differentiation, and migration of B cell precursors. While the ontogeny and cellular composition of the medulla of lymphoid follicles have been well characterized, the cortical compartment was poorly studied, particularly regarding its extracellular matrix (ECM) composition. The cortex grows faster than the medulla, as demonstrated by its higher mitotic index in cortical B cells (15, 49). Functionally, cortical B cells are thought to be the primary source for the peripheral lymphoid organs, entering first into the peripheral blood and later into B cell areas of spleen, thymus and cecal tonsils. In contrast, medullary B cells are a more stable cell population, where their differentiation and migration are influenced by exposure to environmental antigens from the cloacal lumen (26, 50). Our study provides new evidence on how the tenascin-C type of glycoprotein and its interaction with the CXCL12 chemokine regulates cortical B cell migration and lymphoid follicle formation in the chicken BF.

It has previously been described that the cortex of bursal follicles represents a CXCL12-rich environment that guides migration of CXCR4+ B cells towards cortical regions (21), where reticular cells of mesenchymal origin form the scaffold for B cells (30, 51). Here we show that cortical reticular cells express both desmin and vimentin intermediate filaments and are the major source of the CXCL12 chemokine in adult bursal follicles. High concentration of the CXCL12 chemokine in the cortex attracts CXCR4 immunoreactive B cells from the medulla. Double immunofluorescence confirmed a complementary distribution pattern of CXCL12-producing stromal cells and CXCR4^high^ B cells in the outer cortical region, while CXCR4^low/dim^ cells localized near the cortico-medullary border, where tenascin-C was most highly expressed. Unlike the uniformly distributed ECM proteins such as laminin, collagen I, and fibronectin (41), the unique expression pattern of the tenascin-C raised the possibility of a modulatory function during cortical B cell development, particularly in regions of high CXCL12 concentration and active B cell proliferation. Indeed, *in vitro* organ culture and cell migration assays confirmed that tenascin-C functions as an inhibitory matrix component for embryonic B cell migration. Exclusion of tenascin-C during early stages may be essential for enabling migration of B cell precursors into the follicular rudiments. Supporting this hypothesis, our *in ovo* tenascin-C overexpression experiments using Shh-RCAS virus vectors induced ectopic tenascin-C deposition in the bursal mesenchyme and significantly impaired B cell colonization. These findings confirm that tenascin-C is not solely a structural ECM protein but also a negative regulator of chB6+/CXCR4^high^ B cell homing during BF development. As development proceeds, the appearance of tenascin-C expression in the prospective cortical region creates a barrier that inhibits the further migration of B cells into the follicle buds, contributing to the compartmentalization of the BF follicles.

*In situ* hybridization results demonstrate that in the adult BF follicle the cortical mesenchymal reticular cells produce CXCL12, which has been shown to play a critical role in attracting CXCR4-expressing medullary B cells (21, 32). However, the inhibitory effect of tenascin-C on CXCL12 function highlights the complexity of ECM-chemokine interactions in regulating B cell migration. The observed downregulation of CXCR4 in cortical B cells adjacent to the cortico-medullary border and near capillaries suggests a developmental mechanism where B cells exit the CXCL12-rich microenvironment of the cortex to emigrate to peripheral lymphoid organs. Interestingly, a similar process occurs in the murine bone marrow, where CXCL12-abundant reticular (CAR) cells not only attract hematopoietic stem cells but also restrict premature egress of immature B cells through CXCR4 signaling (52). This retention is lifted through CXCR4 downregulation, enabling B cell exit to the periphery. Notably, CAR cells also express tenascin-C, which is critical for HSC maintenance and regeneration following myeloablation, highlighting its conserved role in lymphoid niches (53). According to these molecular similarities, cortical reticular cells of the BF follicles may represent the bursal CAR cells.

Chemokines and growth factors have been previously reported to exert different effects on cell migration based on their solubilized and immobilized forms in several different systems. Shh shows an inhibitory effect on neural crest cell migration only when immobilized to the extracellular matrix (54). Effective affinity maturation of germinal center B cells is dependent on the immobilization of CXCL12 to heparan-sulphate residues of the extracellular matrix in the dark zone (55). Immobilized and soluble chemokines have also been reported to collectively shape the migratory pattern of dendritic cells, where immobilized CCL21 defines a general migratory pathway for adhesive migration, and soluble CCL19 chemokine gradients establish the directionality of movement (56). Switching between adhesion-mediated haptotaxis and non-adhesive migratory patterns is crucial for leukocyte function (57, 58). Our findings show that bursal CXCR4^+^ B‐cells are highly influenced by soluble and surface-immobilized CXCL12. In the presence of tenascin-C and immobilized CXCL12, B cells adopt a polarized phenotype with several long filopodia. The ability of AMD3100 to reverse this effect suggests that CXCL12-CXCR4 signaling is required for tenascin-C-mediated adhesion. Interestingly, similar to its increased expression around cortical blood vessels of the bursa of Fabricius, tenascin-C exhibits an analogous distribution in the mammalian bone marrow, where deposition of tenascin-C is specific to endosteal areas, with high expression around sinusoids. The primary source of tenascin-C are the CXCL12-abundant reticular (CAR) cells, although tenascin-C mRNA has also been detected in endothelial cells (53). Based on this, we propose that the main cellular sources of tenascin-C in the bursal cortex are the CXCL12-expressing mesenchymal reticular cells and the endothelial cells of the cortical capillaries, which explain the high expression level of tenascin-C in the inner cortical region.

One possible mechanism of CXCL12 mediated tenascin-C adhesion is that CXCR4 activation primes inside‐out signaling to integrins (e.g. α4β1 or αvβ3), enhancing cell-matrix adhesion specifically on tenascin-C. When CXCR4 is blocked, integrin activation is reduced, reducing cell adhesion and permitting B cell migration on tenascin-C (59). This tunable switch between adhesion and motility may guide developing and mature B cells within specialized ECM niches of the bursal stroma, ensuring proper exposure to survival and differentiation signals.

Recently, tenascin-C was reported as a critical matrix molecule that impairs CD8^+^ tumor-infiltrating lymphocyte motility in a CXCL12-dependent manner. CXCL12 binds directly to tenascin-C, and pharmacological inhibition of the CXCL12-CXCR4 signaling, using the CXCR4 receptor antagonist AMD3100, releases tumor-infiltrating lymphocytes from their matrix-mediated arrest (47), demonstrating the importance of the CXCL12-tenascin-C interaction in lymphocyte migration. We hypothesize that this evolutionarily conserved “adhesion vs. motility” switch holds developing avian B cells in a specialized microenvironment of the bursal stroma long enough to receive critical survival and differentiation signals until they become receptive to chemokines in the peripheral blood to leave the bursal microenvironment.

In mammals, when B cells egress from bone marrow sinusoids, the cells are predominantly round, migrate in non-amoeboid manner, and their CXCR4 expression is downregulated (52). Only these CXCR4^low/dim^ B cells are able to respond to the increased level of sphingosine-1-phosphate (S1P) - a critical chemoattractant molecule - in the blood serum (60). A recent transcriptomic study also supports the potential role for S1P signalling in regulating chicken CXCR4^low/dim^ B cell egress from embryonic BF and spleen (61).

**Figure 7:**
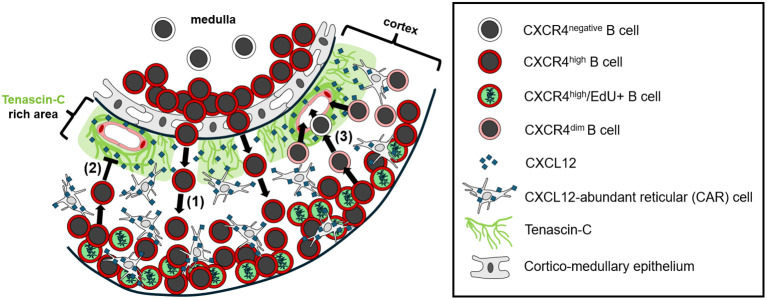
Schematic illustration of B cell migration in the cortex of bursal follicles. 1) CXCR4^high^ medullary B cells migrate towards high concentrations of CXCL12 in the cortex, along tenascin-C free zones between cortical capillaries. CXCL12-abundant reticular (CAR) cells within the cortex establish the high local chemokine concentrations. Soluble CXCL12 interacts with extracellular tenascin-C, forming CXCL12-rich zones around cortical capillaries. 2) Expansion of CXCR4^high^ B cells occurs in outer cortical regions, and CXCL12-rich zones around cortical capillaries prevent premature B cell emigration towards extra-bursal sites. 3) Bursal B cell emigration is dependent on the downregulation of the CXCR4 receptor, allowing cell migration through CXCL12-rich inner cortical zones. CXCR4^low/dim^ B cells reach the periphery through cortical capillaries.

In conclusion, our study identifies tenascin-C as a critical ECM regulator of cortical B cell migration in the avian BF. By modulating the function of CXCL12 and influencing CXCR4 signaling, expression of tenascin-C contributes to proper lymphoid follicle organization and supports the emigration of B cells to peripheral lymphoid tissues. Exclusion of tenascin-C in embryonic BF is crucial for CXCR4^high^ B cell precursor homing, while in later developmental stages tenascin-C expression creates a migration-restrictive environment necessary for follicular compartmentalization. CXCR4 downregulation is required for B cells to leave the cortex and colonize peripheral lymphoid organs (**Figure 7**). Future studies exploring the molecular mechanisms underlying ECM-chemokine interactions will further enhance our understanding of lymphoid organ development and immune cell trafficking. The potential parallels between the BF and mammalian primary lymphoid organs highlight the conserved nature of ECM-chemokine interactions in immune cell development, migration and compartmentalization.

## Data Availability

The original contributions presented in the study are included in the article/supplementary material. Further inquiries can be directed to the corresponding author.
